# Regulation of lymphangiogenesis in the diaphragm by macrophages and VEGFR-3 signaling

**DOI:** 10.1007/s10456-016-9523-8

**Published:** 2016-07-27

**Authors:** Alexandra M. Ochsenbein, Sinem Karaman, Steven T. Proulx, Rhea Goldmann, Jyothi Chittazhathu, Athanasia Dasargyri, Chloé Chong, Jean-Christophe Leroux, E. Richard Stanley, Michael Detmar

**Affiliations:** 1 Institute of Pharmaceutical Sciences, Swiss Federal Institute of Technology, ETH Zurich, Vladimir-Prelog-Weg 3, HCI H303, 8093 Zurich, Switzerland; 2 Department of Developmental and Molecular Biology, Albert Einstein College of Medicine, Yeshiva University, New York, NY 10461 USA

**Keywords:** Lymphatic vessels, Development, Macrophages, VEGFR-3

## Abstract

Lymphatic vessels play important roles in fluid drainage and in immune responses, as well as in pathological processes including cancer progression and inflammation. While the molecular regulation of the earliest lymphatic vessel differentiation and development has been investigated in much detail, less is known about the control and timing of lymphatic vessel maturation in different organs, which often occurs postnatally. We investigated the time course of lymphatic vessel development on the pleural side of the diaphragmatic muscle in mice, the so-called submesothelial initial diaphragmatic lymphatic plexus. We found that this lymphatic network develops largely after birth and that it can serve as a reliable and easily quantifiable model to study physiological lymphangiogenesis in vivo. Lymphangiogenic growth in this tissue was highly dependent on vascular endothelial growth factor receptor (VEGFR)-3 signaling, whereas VEGFR-1 and -2 signaling was dispensable. During diaphragm development, macrophages appeared first in a linearly arranged pattern, followed by ingrowth of lymphatic vessels along these patterned lines. Surprisingly, ablation of macrophages in colony-stimulating factor-1 receptor (*Csf1r*)-deficient mice and by treatment with a CSF-1R-blocking antibody did not inhibit the general lymphatic vessel development in the diaphragm but specifically promoted branch formation of lymphatic sprouts. In agreement with these findings, incubation of cultured lymphatic endothelial cells with conditioned medium from P7 diaphragmatic macrophages significantly reduced LEC sprouting. These results indicate that the postnatal diaphragm provides a suitable model for studies of physiological lymphangiogenic growth and maturation, and for the identification of modulators of lymphatic vessel growth.

**Electronic supplementary material:**

The online version of this article (doi:10.1007/s10456-016-9523-8) contains supplementary material, which is available to authorized users.

## Introduction

Lymphatic vessels play important roles in physiological tissue fluid homeostasis, immune surveillance and dietary lipid uptake, as well as in a range of pathological conditions including chronic inflammation, wound healing and cancer progression [15, 37]. Despite these important roles, only few mediators of lymphatic vessel development and function have been identified. Among those, vascular endothelial growth factor receptor (VEGFR)-3 and its ligands VEGF-C and VEGF-D represent the major lymphangiogenic signaling system. VEGFR-2 signaling might also promote lymphangiogenesis during metastasis formation in the lymph node [12] and during lymphatic vessel enlargement in inflamed skin [21, 38], but the role of VEGFR-2 during developmental lymphangiogenesis is less clear.

The lymphatic vascular system starts to develop around midgestation by the budding of Prox-1-positive lymphatic endothelial cells (LECs), which also express VEGFR-3, from the cardinal vein. These cells then migrate toward a VEGF-C gradient and form a lymphatic vessel plexus, which becomes remodeled from embryonic day (E) 14.5 on, and matures until postnatal time points [28, 31, 40]. Maturation processes include the differentiation of lymphatic vessels into capillaries and collecting lymphatic vessels that are characterized by sparse smooth muscle cell coverage, down-regulation of LYVE-1 expression, and valves that prevent the backflow of lymph [23, 30]. Importantly, the time course of lymphatic vessel development can vary in different organs, and for many organs, the exact time course is still unknown.

There are few in vivo models that allow a quantitative characterization of physiological lymphangiogenesis. The two most widely used models study the development of superficial lymphatic vessels in the tail dermis [39] and the development of collecting lymphatic vessels in the mesentery [27]. A potential disadvantage of both models is that lymphatic vessels in adults cannot be studied with whole-mount approaches and that the study of lymphatic vessel sprouting in vivo remains rather difficult.

The role of macrophages in pathological lymphangiogenesis has been thoroughly investigated (reviewed in [11]), and macrophages can serve in these settings as sources of pro-lymphangiogenic growth factors such as VEGF-C and VEGF-D [3, 18, 19]. In contrast, the role of macrophages in developmental lymphangiogenesis is less clear. Macrophage depletion resulted in reduced lymphatic vessel branching in the ears and the trachea of newborns [20], whereas it increased the lymphatic vessel diameter in embryonic skin and led to hypoplastic jugular lymph sacs [10]. These differing results suggest a tissue-dependent role of macrophages in developmental lymphangiogenesis.

The aim of the present study was to investigate the developmental time course of lymphatic vessel formation on the pleural side of the diaphragm, the so-called submesothelial initial diaphragmatic lymphatic plexus [26], and its potential molecular and cellular mediators. We found that development of the diaphragmatic lymphatic plexus largely occurs postnatally and that it represents a sensitive model to study the regulation of lymphangiogenesis in vivo. Normal development was dependent on VEGFR-3 signaling but largely independent of VEGFR-1 or VEGFR-2 signaling. Studies in colony-stimulating factor-1 receptor (*Csf1r*)-deficient mice and antibody depletion of CSF-1R surprisingly revealed that macrophages are dispensable for the general development of the diaphragmatic lymphatic network but that they restrain the formation of lymphatic vessel branches by secreted factors.

## Materials and methods

### In vivo experiments

Mice were bred and housed in the animal facility of ETH Zurich. Experiments were performed in accordance with the animal protocol 131/2014 approved by the local veterinary authorities (Kantonales Veterinäramt Zürich). *Prox*-*1* GFP mice [5] were provided by Dr. Young-Kwon Hong (Keck School of Medicine, USD, California). K14-VEGFR-3-Fc mice (Mäkinen et al., 2001) were provided by Dr. Kari Alitalo (Institute of Biomedicine, Biomedicum, University of Helsinki). *Csf1r*−/− mice [6] were obtained by mating heterozygotes that had been backcrossed for 10 generations to the FVB/NJ background. For the in utero VEGFR blockade, pregnant C57BL/6 female mice were injected intraperitoneally (i.p.) with 800 µg blocking antibodies against VEGFR-1 (mF1), VEGFR-2 (DC101) or VEGFR-3 (mF4-31C1) (kindly provided by Dr. Bronek Pytowski, ImClone Systems/Eli Lilly), or with control rat IgG (Sigma-Aldrich) at E16.5 and E18.5, and pups were killed at postnatal day (P) 5. To deplete macrophages with an antibody-mediated approach, pups were injected (i.p.) with the purified neutralizing rat anti-mouse CSF-1R monoclonal antibody AFS98 [36] or rat IgG (Sigma) (36.5–50 mg/kg bodyweight) at P0, P1, P3 and P5 and then killed at P7.

### Immunostaining

Tail skin and diaphragms were fixed in fresh 4 % paraformaldehyde (PFA) for 2 h and blocked at room temperature for 3 h in phosphate-buffered saline (PBS) containing 5 % donkey serum, 1 % bovine serum albumin (BSA) and 0.1 % Triton-X. The following antibodies were incubated overnight: Rabbit anti-LYVE-1 (AngioBio), goat anti-LYVE-1 (R&D Systems), rat anti-CD31 (BD Biosciences Pharmingen), goat anti-CD206 (R&D Systems), mouse anti-smooth muscle actin (Sigma-Aldrich), rat anti-CD68 (Abcam), goat anti-Prox-1 (R&D Systems), rabbit anti-Prox-1 (kind gift of Dr. Kari Alitalo), rat anti-F4/80 (Abcam) and rat anti-CSF-1R (AFS98 hybridoma antibody). After an intense washing step, tissues were incubated for 2 h at room temperature with Alexa Fluor 488, 594 or 647 nm-conjugated secondary antibodies (Invitrogen) and mounted in Vectashield (Vector) for confocal imaging. Whole-mount z-stack images were acquired with a LSM 710 FCS confocal microscope and ZEN software (Zeiss) and processed with Photoshop CS5 (Adobe) and ImageJ software (NIH).

### Morphological analyses of diaphragmatic lymphatic vessels

To analyze the lymphatic vessels on the pleural side of the diaphragmatic muscle, a diaphragm segment was imaged by taking partially overlapping confocal images (using a 10× 0.3NA EC Plan-Neofluar objective) from the thorax wall to the central tendon in the crural diaphragm region. Pictures were merged using Photoshop software, and the merged diaphragm segments were analyzed with ImageJ to quantify branch numbers, average branch length and relative LYVE-1-positive area. The diameter was calculated by dividing the LYVE-1+ area by the total vessel length. Lymphatic vessels on the central tendon were excluded from the analyses. Photoshop was used to count lymphatic vessel loops.

### Hybridoma cell culture and anti-CSF-1R (AFS98) antibody purification

The AFS98 hybridoma cell line was a kind gift from Dr. Shin-Ichi Nishikawa, RIKEN Center for Developmental Biology, Kobe, Japan. AFS98 cells were cultured at 37 °C with 5 % CO_2_ in serum-free Hybridoma-SFM medium (Gibco, Life Technologies). The anti-CSF-1R antibody was purified from pooled medium with a Profinia Protein Purification device (Bio-Rad), using a protein G column (GE Healthcare Life Sciences) and treated with an endotoxin removing gel (Detoxi-Gel, Thermo Scientific) to remove possible endotoxin contamination.

### Lymphatic sprouting assay

Macrophage-conditioned medium was obtained by isolating CD11b+ cells from diaphragms of P7 WT pups (*n* = 6) by MACS (Miltenyi Biotech), and culturing them in 24 wells for 48 h in RPMI + 1 % FBS medium. Sprouting assays were performed largely as described [32]. Gelatin-covered cytodex microcarrier beads (Sigma) were coated with lymphatic endothelial cells at a 1:40 bead-to-cell ratio and incubated at 37 °C. LEC-covered beads were labeled with Cell Tracker Green (CTG, Invitrogen) and embedded into a 1 mg/mL type I collagen solution (Advanced Biomatrix) in black 96-well clear-bottom plates (Costar). The collagen was left to polymerize before incubation with a medium consisting of a 1:1 mixture of EBM + 20 % FBS and macrophage-conditioned medium or RPMI + 1 % FBS medium, with or without the addition of 80 ng/ml recombinant human VEGF-A165 (R&D Systems) and 80 ng/ml bFGF (R&D Systems). Samples were fixed in 2 % paraformaldehyde (Sigma). The sprouts were imaged with an automated epifluorescence microscope ImageXpress micro (Molecular Devices, California, USA), equipped with a 4 × 0.2 NA Plan Apo objective (Nikon, Tokyo, Japan), a Lumencor Spectra X light engine (Lumencor, Oregon, USA), a Photometrics CoolSNAP HQ digital CCD camera (Photometrics, Arizona, USA) and a 488-nM excitation FITC filter set and controlled with the MetaXpress 5 software (Molecular Devices). The number of sprouts per bead was analyzed manually per 96-well image.

### Preparation and characterization of PEGylated fluorescent beads

Yellow–green fluorescent polystyrene amine-modified microspheres (FluoSpheres, Molecular Probes Europe BV, Netherlands) of 1 μm diameter (2 mg, 12 nmol) were washed with water twice by centrifugation and then resuspended in 1 ml of DMSO and sonicated for 10 min in a bath sonicator. Methoxy-PEG 5000-NHS (JenKem Technology, Allen, TX, USA) (1 mg, 200 nmol) was added, and the mixture was left to react overnight at room temperature under stirring. After reaction, the mixture was diluted in 5 ml of water and centrifuged (4000×*g*, 15 min, 15 °C) to remove the DMSO. The particles were washed 4 times by centrifugation/resuspension in water to remove the excess of unreacted polymer. Finally, they were resuspended in 1 ml of PBS, resulting in a final concentration of 2 mg/ml. The microspheres were characterized in terms of ζ-potential by laser Doppler anemometry using a DelsaNano C Particle Analyzer (Beckman Coulter, Krefeld, Germany). The ζ-potential value of −1 mV indicated adequate PEGylation of the beads.

### Lymphatic drainage assay

To determine the lymphatic drainage of peritoneal fluid into the draining lymph nodes via diaphragmatic lymphatic vessels, P7 pups were injected i.p. with 10 µl of 0.2 mg/ml PEGylated 1-µm-diameter yellow–green fluorescent microspheres in PBS. At 1 h after injection, beads were visualized in situ within draining lymph nodes using a Zeiss StereoLumar.V12 stereomicroscope (Carl Zeiss, Oberkochen, Germany) equipped with an EMCCD camera (Evolve eXcelon, Photometrics, Tucson, AZ), a high-powered light-emitting diode (LED) system with illumination at 470 nm (CoolLED, Andover, UK) and specific filters for FITC (Zeiss). For FACS analysis, after 1 h, the draining mediastinal lymph nodes were collected and passed through a 40-µm cell strainer (Invitrogen) to generate single-cell suspensions. FACS analyses were performed using a FACSAria and the FACSDiva software (BD Biosciences), and beads per lymph nodes were determined. Pictures were taken with a Zeiss StereoLumar.V12 stereomicroscope.

### Statistical analyses

Data are shown as mean ± SD, and two-tailed Student’s *t* test or one-way ANOVA and the Dunnett’s multiple comparison tests were used to compare two or more groups, respectively. Statistical significance is indicated by asterisks: *p < 0.05, **p < 0.01, ***p < 0.001. Statistical significance was set as *p* < 0.05.

## Results

### Lymphatic vessels on the pleural side of the diaphragmatic muscle develop and mature postnatally

**Fig. 1:**
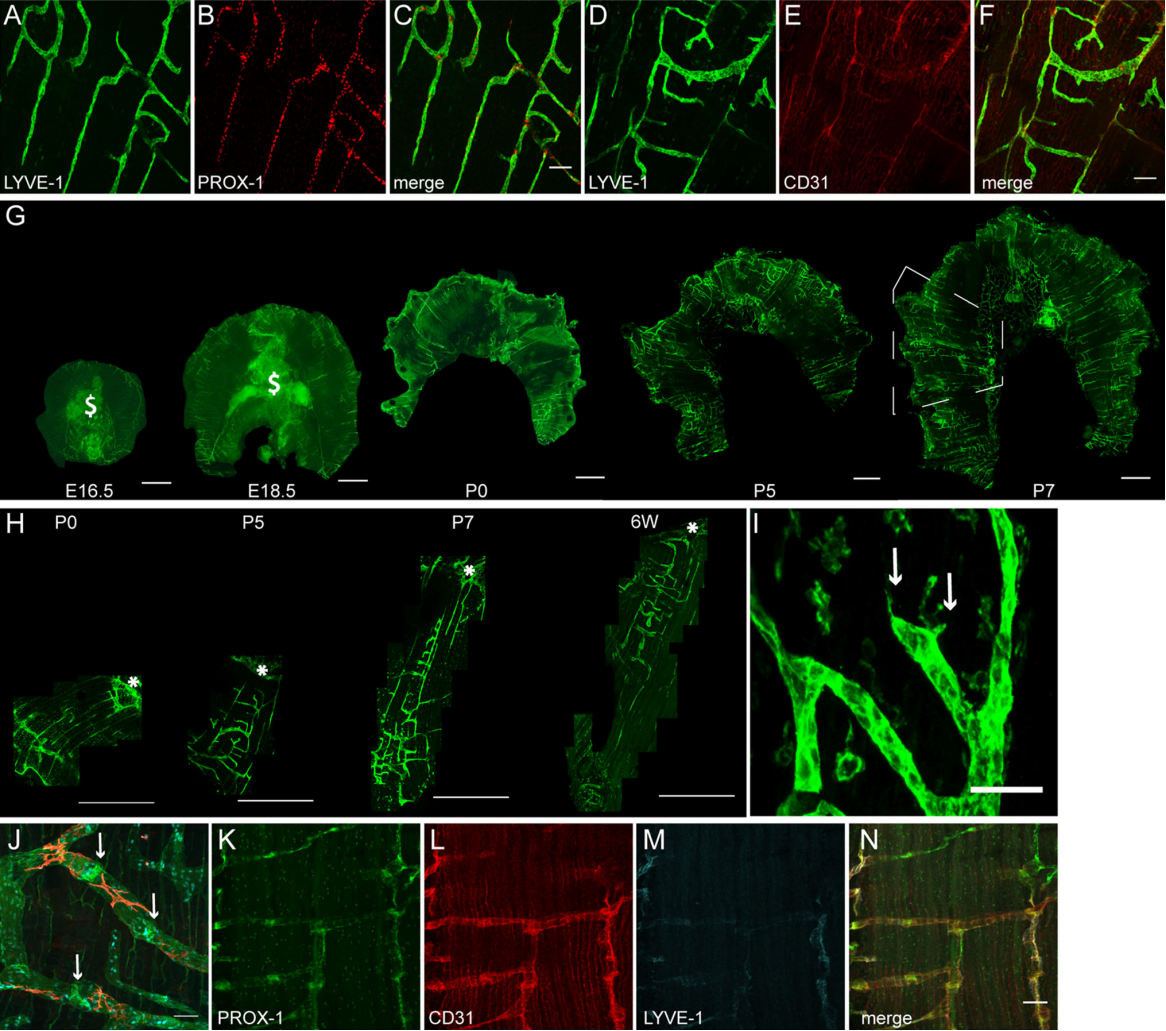
Lymphatic vessels on the pleural side of the diaphragmatic muscle develop and mature postnatally. Whole mounts of C57BL/6 mice show that on the pleural side of the diaphragmatic muscle, LYVE-1-positive vessel structures (*green*) co-stain for Prox-1 (*red*) at P7 (**a**–**c**) and for CD31 (*red*) at P5 (**d**–**f**). Wide-field images of LYVE-1 whole mounts demonstrate few LYVE-1-positive vessel structures at E16.5 and E18.5 and an expansion of the vessel network from P0 to P7 (**g**). $ mark unspecific staining of liver tissue. *Dotted lines mark* the area of the diaphragm that is used for whole-mount imaging and quantifications (**g**). Merged confocal images of lateral diaphragm segments stained for LYVE-1 show the expansion of the vessel plexus starting from P0 to 6 W (**h**). *Stars mark* the central tendon region. High-magnification confocal images allow the visualization of diaphragmatic lymphatic vessel sprouts at P7 (**i** *arrows*). Prox-1 (*cyan*), α-SMA (*red*) and CD31 (*green*) whole-mount stainings show valves (*arrows*) and SMC coverage of lymphatic vessels, located close to the thorax wall (**j**). CD31 (*red*) and LYVE-1 (*cyan*) diaphragm whole mounts of a 6-week-old *Prox*-*1* GFP mouse show LYVE-1 down-regulation on diaphragmatic lymphatic vessels located close to the thorax wall (**K-N**). *Scale bars* **a**–**f** 100 µm, **g** and **h** 1 mm, **i** 50 µm, **j** 100 µm, **k**–**n** 50 µm. (Color figure online)

To investigate whether the submesothelial initial diaphragmatic lymphatic plexus might be suitable as a reliable and quantifiable model of developmental lymphangiogenesis, we first stained diaphragm whole mounts from newborn C57BL/6 mice for the lymphatic vessel markers LYVE-1 (Fig. 1a, d) and Prox-1 (Fig. 1b), as well as for the pan-endothelial marker CD31 (Fig. 1e). LYVE-1+ vessels also expressed Prox-1 (Fig. 1c) and CD31 (Fig. 1f), allowing the visualization of individual lymphatic vessels and branches, a feature required for the detailed analysis and quantification of lymphatic vessel growth.

We next studied the lymphangiogenic processes on the pleural side of the diaphragmatic muscle by analysis of whole mounts at time points E16.5, E18.5, P0, P5 and P7. Wide-field images showed that only very few LYVE-1+ lymphatic vessels were present close to the thorax wall at E16.5, with slightly more vessels apparent at E18.5 (Fig. 1g). At P0, the lymphatic vessels grew radially from the thorax wall toward the central tendon. At P5, the radial growth of lymphatic vessels was more pronounced, and at P7, a fully developed lymphatic vessel network was visible with lymphatic vessels spanning from the thorax wall to the central tendon as individual or branched vessels (Fig. 1g). At E16.5 and E18.5, LYVE-1 was also expressed in liver tissue that was attached to the diaphragm at those time points (Fig. 1g, marked by $). For further high-resolution confocal imaging and quantification, lateral segments of the pleural diaphragmatic muscle were chosen as indicated in Fig. 1g. Confocal images of lateral segments stained for LYVE-1 showed the expansion of the lymphatic vessel network from P0 to P5 and P7 in more detail (Fig. 1h) and also allowed the visualization of lymphatic vessels on the pleural side of the diaphragm at 6 weeks of age (Fig. 1h). High-magnification images revealed lymphatic vessel sprouts at P7 (Fig. 1i, arrows). At 4 weeks of age, the lymphatic vessels showed characteristics of maturation such as smooth muscle cell coverage and the presence of valves (Fig. 1j; arrow). To investigate whether LYVE-1 might be downregulated on mature lymphatic vessels at later time points, diaphragm whole mounts of 6-week-old *Prox*-*1* GFP transgenic mice (Fig. 1k) were stained for CD31 (red, Fig. 1l) and LYVE-1 (cyan, Fig. 1m). We found that LYVE-1 expression on the Prox-1 and CD31-positive lymphatic vessels located close to the thorax wall was weaker than at earlier time points (Fig. 1n), demonstrating that the lymphatic vessels on the pleural side of the diaphragmatic muscle also partially show this maturation phenotype.

### Diaphragmatic lymphatic vessel growth is VEGFR-3 dependent

**Fig. 2:**
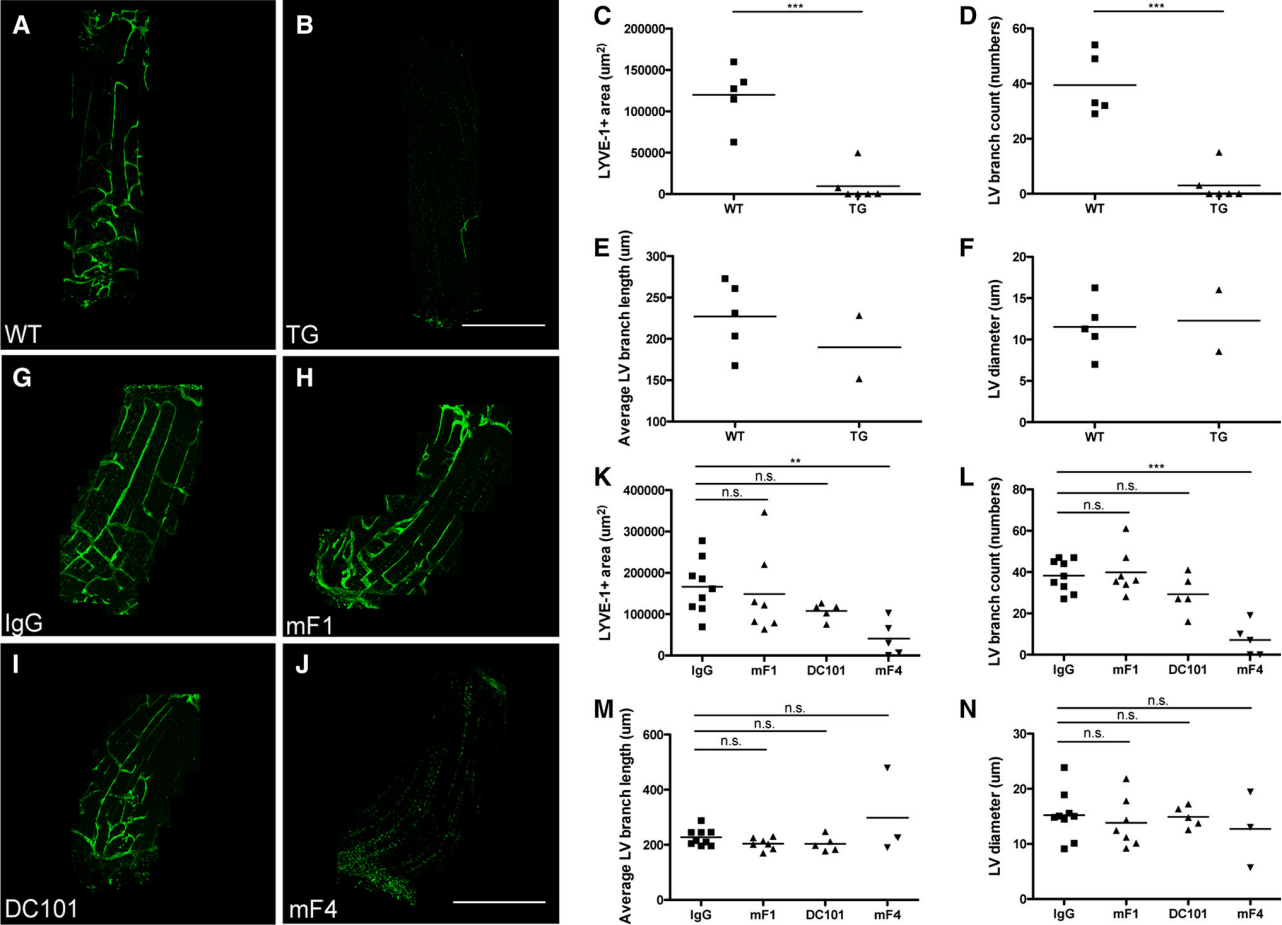
Diaphragmatic lymphatic vessel growth is VEGFR-3 dependent. Segments of diaphragm whole mounts stained for LYVE-1 of P7 WT (**a**) and K14-VEGFR-3-Fc transgenic (TG) littermates (**b**). Quantification of diaphragmatic lymphatic vessel (LV) development of WT and K14-VEGFR-3-Fc transgenic (TG) pups (**c**–**f**). *Dots* represent mean values per mouse, and lines indicate the group means. As only 2 out of 6 diaphragm segments of TG pups had detectable vessels, no statistical analysis was performed for the parameters average LV branch length (**e**) and LV diameter (**f**). Segments of LYVE-1-stained pleural diaphragm whole mounts of P5 pups treated with IgG control (**g**) or antibodies blocking VEGFR-1 (mF1) (**h**), VEGFR-2 (DC101) (**i**) or VEGFR-3 (mF4) (**j**) in utero at E16.5 and E18.5. Quantification of diaphragmatic lymphatic vessel development of VEGFR-blocking antibody-treated pups (**k**–**n**). *Dots* represent mean values per mouse, and lines indicate the group means. *Scale bars* 1 mm. *p < 0.05; **p < 0.01; ***p < 0.001

We next investigated the role of VEGFR-3 in the development of lymphatic vessels on the pleural side of the diaphragm. Diaphragm whole mounts obtained from K14-VEGFR-3-Fc transgenic mice and wild-type (WT) littermates were stained for LYVE-1 at P7. K14-VEGFR-3-Fc transgenic mice express a soluble form of VEGFR-3 constitutively, which acts as a decoy receptor for its ligands VEGF-C and VEGF-D, under control of the K14 promoter. Merged confocal images of the diaphragm segments revealed an almost complete absence of lymphatic vessels in the diaphragms of K14-VEGFR-3-Fc mice (Fig. 2a, b). To further quantify this phenotype, we measured the LYVE-1+ area, the average lymphatic branch length and the average lymphatic vessel diameter. K14-VEGFR-3-Fc mice had a significant, 92 % decrease in the LYVE-1+ area (WT: 0.12 ± 0.036 mm^2^, TG: 0.01 ± 0.2 mm^2^, *n* = 5–6 per group; *p* = 0.00086, Fig. 2c), and a 92 % decrease in branch numbers (WT: 39.4 ± 11.28, TG: 3 ± 6, *n* = 5–6 per group; *p* = 0.00067, Fig. 2d) compared to WT controls. In the few cases (2 out of 6 K14-VEGFR-3-Fc pups) where a few lymphatic vessels were detectable, the average branch length and diameter remained unchanged compared to WT controls (Fig. 2e, f).

Since both VEGFR-2 and VEGFR-3 have been implicated in physiological and pathological lymphatic vessel formation [9, 13, 38, 41], we next aimed to dissect the individual influence of different VEGFRs during lymphatic development in the diaphragm. Pregnant C57BL/6 female mice were injected i.p. with blocking antibodies against VEGFR-1 (mF1), VEGFR-2 (DC101) or VEGFR-3 (mF4), or IgG control at E16.5 and E18.5, when the diaphragmatic lymphatic plexus in the embryos is just about to form, and analyses of diaphragmatic lymphatic vessels were performed at P5. Confocal images of LYVE-1-stained diaphragm segments revealed an almost complete inhibition of lymphatic vessel growth in diaphragms of mF4-treated pups, as compared to IgG controls (Fig. 2g, j). In contrast, treatment with mF1 or DC101 did not lead to any obvious alterations of the lymphatic vessel phenotype on the pleural side of the diaphragmatic muscle (Fig. 2g–j). Quantitative analyses revealed that systemic blockade of VEGFR-3 significantly reduced the LYVE-1+ area by 75 % (IgG: 0.167 ± 0.065 mm^2^, mF4: 0.041 ± 0.043 mm^2^, *n* = 5–9 pups per group; *p* ≤ 0.01, Fig. 2k) and the branch numbers by 81 % (IgG: 38.3 ± 7.8, mF4: 7.2 ± 5, *n* = 5–9 pups per group; *p* ≤ 0.001, Fig. 2l) compared to IgG controls. With blockade of VEGFR-2, there was a nonsignificant trend toward reduced LYVE-1+ area and branch counts, whereas blockade of VEGFR-1 had no discernable impact on these parameters (Fig. 2k–n). The average length of lymphatic vessel branches and the average lymphatic vessel diameter were comparable in all groups.

### VEGFR-3 blockade leads to similar lymphatic vessel phenotypes in the tail dermis and on the pleural diaphragm side

**Fig. 3:**
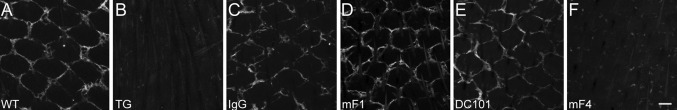
VEGFR-3 blockade leads to similar lymphatic vessel phenotypes in the tail dermis and on the pleural diaphragm side. LYVE-1 whole mounts of tail dermis of P7 WT (**a**) and K14-VEGFR-3-Fc transgenic littermates (**b**). LYVE-1 whole mounts of tail dermis of P5 pups treated with IgG (**c**) or blocking antibodies against VEGFR-1 (mF1) (**d**), VEGFR-2 (DC101) (**e**), or VEGFR-3 (mF4) (**f**) in utero at E16.5 and E18.5. VEGFR-3 blockade leads in both approaches to an almost complete inhibition of the lymphangiogenic process, whereas VEGFR-1 and -2 blockades have no obvious effect. *Scale bar* 100 µm

To investigate whether similar changes as in the diaphragm model could be seen in another model for developmental lymphangiogenesis, we studied the superficial lymphatic vessel plexus in the tail dermis. Confocal images of LYVE-1 whole mounts of the tail dermis of K14-VEGFR-3-Fc transgenic mice and WT littermates showed that the transgene expression almost completely inhibited the development of the superficial lymphatic plexus at P7 (Fig. 3a, b). LYVE-1 whole mounts of tail dermis after in utero treatment with IgG or with VEGFR-1 (mF1), VEGFR-2 (DC101) or VEGFR-3 (mF4)-blocking antibodies revealed that only blockade of VEGFR-3 inhibited lymphangiogenesis at P5 (Fig. 3c–f), similar to the findings in the diaphragm.

### Macrophages are closely aligned with lymphatic vessels and their sprouting tips, and emerge before lymphatic vessels

**Fig. 4:**
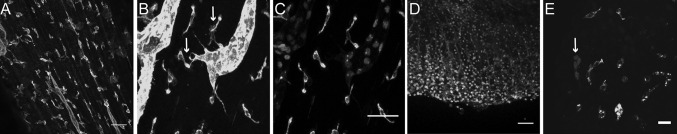
Macrophages are closely aligned with lymphatic vessels and their sprouting tips and develop before lymphatic vessels. Diaphragm whole mounts stained for LYVE-1 (*red*), CD206 (*cyan*) and F4/80 (*green*) at P8 show macrophages aligned in parallel to lymphatic vessels (**a**). P7 diaphragm whole mounts show that LYVE-1 (*green*)- and CD206 (*cyan*)-positive macrophages have close contact with the sprouting tip of LYVE-1-positive lymphatic vessels (**b**, *arrows*), but they are not positive for Prox-1 (*red*) (**c**). E14.5 diaphragm whole mounts stained for CD206 (*red*), LYVE-1 (*cyan*) and CD31 (*green*) show the presence of CD206- and LYVE-1-positive macrophages but no LYVE-1-positive vessel structures (**d**). At E14.5, the few Prox-1+ lymphatic endothelial cells (*cyan*) do not co-stain for CD68 (*green*) (**e**, *arrow*). *Scale bars* **a** 50 µm, **b** and **c** 50 µm, **d** 100 µm, **e** 20 µm. (Color figure online)

Macrophages have been reported to share an intimate association with lymphatic vessels [10, 42] and to drive lymphangiogenesis in many tumor and inflammation settings [11]. To investigate whether macrophages are also important for the normal lymphatic vessel development in the diaphragm, we first investigated the localization of macrophages and lymphatic vessels. By whole-mount staining, we detected an alignment of F4/80 (green)- and CD206 (cyan)-positive macrophages with LYVE-1 (red)-positive growing lymphatic vessels on the pleural side of the diaphragmatic muscle at P7 (Fig. 4a). Intriguingly, high-magnification confocal images showed a close interaction of sprouting lymphatic vessels (LYVE-1 green, Prox-1 red) and macrophages (cyan) at P8 (Fig. 4b, c). We did not observe Prox-1-positive macrophages in the diaphragm, suggesting that macrophages do not acquire a lymphatic endothelial cell signature during diaphragm development (Fig. 4c). LYVE-1- and CD206-positive macrophages were already present in E14.5 diaphragms when CD31- and LYVE-1-positive vessel structures were not yet detectable (Fig. 4d). Again, also at this time point, the few Prox-1-positive LECs (cyan) that were detectable did not co-stain for CD68 (green, Fig. 4e).

### Colony-stimulating factor-1 receptor-positive macrophages negatively regulate lymphatic vessel branching on the pleural side of the diaphragm

**Fig. 5:**
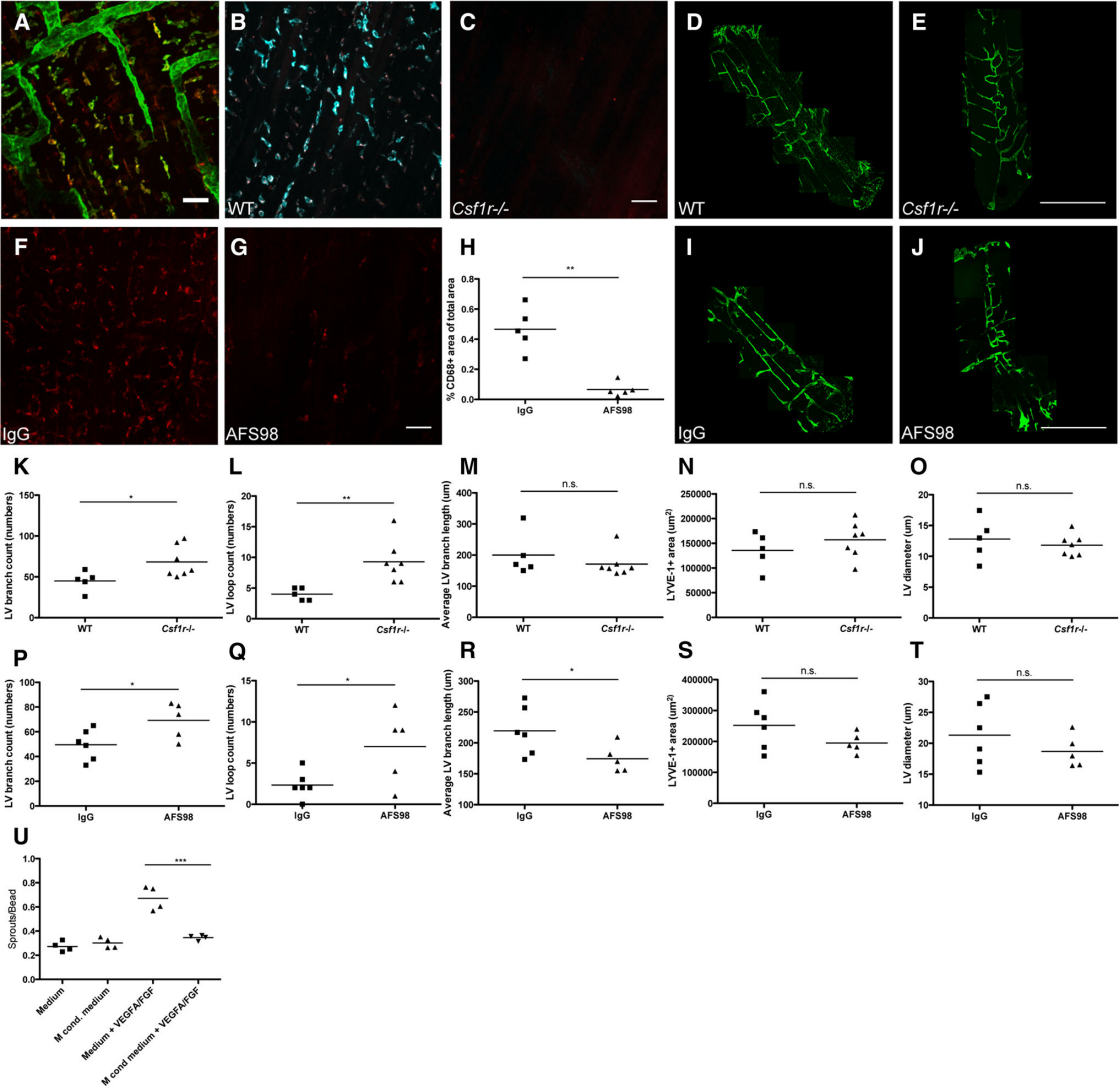
Colony-stimulating factor-1 receptor-positive macrophages negatively regulate lymphatic vessel branching on the pleural side of the diaphragm. P6 diaphragm whole mounts show CSF-1R and LYVE-1 double-positive macrophages in proximity to LYVE-1-positive lymphatic vessels (**a**). Compared to WT littermates (**b**), CD206 (*cyan*)- and CD68 (*red*)-stained whole mounts of diaphragms of P7 *Csf1r*−/− pups show depletion of macrophages (**c**). Segments of pleural diaphragm LYVE-1 whole mounts of P7 WT (**d**) or *Csf1r*−/− pups (**e**). CD68-stained whole mounts of IgG (**f**)- or AFS98 (**g**)-treated pups show depletion of macrophages on the pleural side of the diaphragmatic muscle by AFS98 treatment. Quantification of the CD68-positive area per visual field in the diaphragm (**h**). *Dots* represent mean values per mouse, and lines indicate the group means. Quantification of diaphragmatic lymphatic vessel development of *Csf1r*−/− pups shows a significant increase in branches and lymphatic loops compared to the WT controls (**k**–**o**). *Dots* represent mean values per mouse, and lines indicate the group means. Segments of pleural diaphragm LYVE-1 whole mounts of P7 IgG (**i**)- or AFS98-treated pups (**j**). Quantification of diaphragmatic lymphatic vessel development of P7 AFS98-treated pups shows a significant increase in branches, lymphatic loops and a significant decrease in average branch length compared to the IgG controls (**p**–**t**). *Dots* represent mean values per mouse, and *lines* indicate the group means. Quantifications of LEC sprouts per bead show that isolated P7 diaphragmatic macrophage-conditioned medium significantly decreased VEGF-A/FGF-induced LEC sprouting (**u**). *Dots* represent mean values per well, and *lines* indicate the group means. *Scale bars* **a** 50 µm, **b** and **c** 50 µm, **d** and **e** 1 mm, **f** and **g** 50 µm, **i** and **j** 1 mm. *p < 0.05; **p < 0.01; ***p < 0.001. (Color figure online)

To investigate the biological role of macrophages during lymphatic vessel development in the diaphragm, we next studied the lymphangiogenic process after systemic inhibition of CSF-1R signaling. CSF-1R signaling regulates tissue macrophage homeostasis, and its absence leads to macrophage depletion [4, 6]. We found that CSF-1R (red) was expressed by LYVE-1+ macrophages but not by lymphatic vessels at P6 (Fig. 5a). The effects of macrophage depletion were assessed by immunofluorescent stainings of diaphragms, tail skin and back skin for the macrophage markers F4/80, CD68, CD11b, CD206, and for LYVE-1 (Fig. 5b, c, f, g and Supplementary Material S1A–D, M–P). Merged confocal images of diaphragm segments stained for LYVE-1 showed an almost complete depletion of LYVE-1+ macrophages and increased lymphatic vessel branches in *Csf1r*−/− pups at P7 (Fig. 5d, e). Quantification revealed that *Csf1r*−/− mice had significantly increased (+51 %) lymphatic vessel branch counts per analyzed diaphragm segment (WT: 45.00 ± 12.02, *Csf1r*−/−: 68.14 ± 19.36, *n* = 5–7 pups per group; *p* = 0.029, Fig. 5k) and a significantly increased number of lymphatic vessel loops per analyzed diaphragm segment (+132 %; WT: 4.00 ± 1.00, *Csf1r*−/−: 9.29 ± 3.45, *n* = 5–7 pups per group; *p* = 0.006, Fig. 5l) compared to wild-type controls. The LYVE-1+ area, average branch length and vessel diameter remained unchanged (Fig. 5m–o).

Similar results were obtained after antibody-mediated blockade of CSF-1R. Intraperitoneal injections of AFS98, a CSF-1R-blocking antibody, at P0, P1, P3 and P5 significantly decreased the CD68+ area in the diaphragm compared to IgG control (−86 %; IgG: 0.47 ± 0.15 %, AFS98: 0.07 ± 0.05 %, *n* = 5 pups per group, *p* = 0.0023, Fig. 5f–h), as well as LYVE-1+ macrophages (Fig. 5i, j) at day P7. The treatment also significantly increased the number of lymphatic vessel branches per analyzed diaphragm segment (+40 %; IgG: 49.5 ± 12.34, AFS98: 69.2 ± 14.55, *n* = 5–6 pups per group, *p* = 0.038, Fig. 5p) and the loop formation by lymphatic vessels (+200 %, IgG: 2.34 ± 1.63, AFS98: 7 ± 4.42, *n* = 5–6 pups per group, *p* = 0.039, Fig. 5q). The average lymphatic vessel length was significantly reduced after CSF-1R blockade (−20 %; IgG: 219.39 ± 39.23 µm, AFS98: 174.31 ± 22.35 µm, *n* = 5–6 pups per group, *p* = 0.0495, Fig. 5r). The LYVE-1+ area and lymphatic vessel diameters remained unchanged (Fig. 5s, t).

No significant differences were detectable in the LYVE-1-positive area and the number of lymphatic vessel branches per lymphatic ring structure in the tail dermis (Supplementary Material Fig. S1E–H) and the number of lymphatic vessel branches and lymphatic valves in mesenteric lymphatic vessels of *Csf1r*−/− mice (Supplementary Material Fig. S1I–L).

To obtain deeper mechanistic insights into how macrophages might regulate lymphatic vessel branching, we next used LEC sprouting assays [32]. We found that conditioned media obtained from isolated P7 diaphragmatic macrophages significantly reduced the VEGF-A/bFGF-induced LEC sprouting (medium + VEGF-A/FGF: 0.671 ± 0.044 sprouts/bead; macrophage-conditioned medium + VEGF-A/FGF: 0.346 ± 0.021 sprouts/bead, *n* = 4 wells per group, *p* = 0.000691, Fig. 5u). This finding indicates that diaphragmatic macrophages inhibit LEC sprouting by secreted factors.

### Macrophage depletion does not impair lymphatic vessel drainage function

**Fig. 6:**
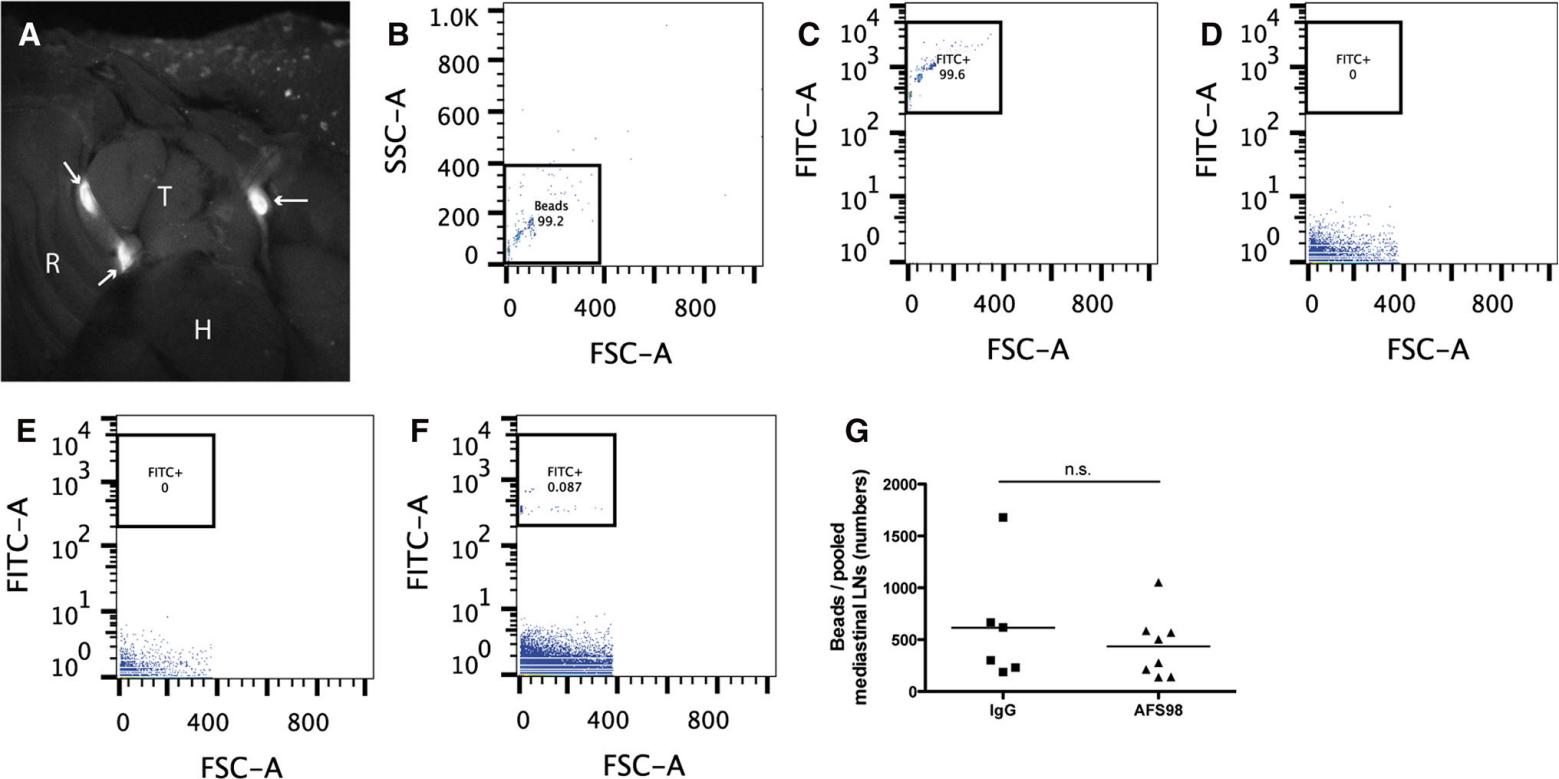
Macrophage depletion does not impair lymphatic vessel drainage function. Stereomicroscope image showing fluorescent microspheres within mediastinal lymph nodes (**a**, *arrows*) in exposed thorax 1 h after i.p. injection of microspheres into P8 pup. *T* thymus, *H* heart, *R* rib cage. FACS analysis in the FSC/SSC (**b**) and FITC/FSC (**c**) channels. FACS analysis of green–yellow fluorescent microspheres in PBS in mediastinal lymph nodes of uninjected pups (**d**), and in non-draining inguinal lymph nodes (**e**) and in draining mediastinal lymph nodes (**f**) 1 h after i.p. injection of microspheres into WT P8 pups. Quantification by FACS showed that there was no difference in drainage of fluorescent microspheres to pooled mediastinal lymph nodes 1 h after i.p. injection of microspheres into rat IgG- or AFS98-treated P7 pups (**g**)

Diaphragmatic lymphatic vessels represent a central route for the drainage of peritoneal fluid [1, 35], draining molecules and fluid to the mediastinal lymph nodes in adult mice [19]. We next investigated if macrophage depletion might also have an effect on diaphragmatic lymphatic vessel drainage. P8 pups were injected i.p. with 10 µl of PEGylated yellow–green fluorescent microspheres in PBS, and the draining mediastinal lymph nodes were analyzed after 1 h. Stereomicroscopic images showed that the mediastinal lymph nodes contained microspheres (Fig. 6a, arrows). Quantitative FACS analysis revealed that microspheres are quantifiable in a PBS solution (Fig. 6b, c) and that microspheres were detectable in mediastinal lymph nodes (Fig. 6f) after i.p. injection, but not in non-draining inguinal lymph nodes (Fig. 6e) or in mediastinal lymph nodes of uninjected pups (Fig. 6d). After treatment with AFS98, FACS analysis showed no significant differences of microsphere content in the mediastinal lymph nodes, as compared to control pups (Fig. 6g).

## Discussion

Our study reveals that the submesothelial initial diaphragmatic lymphatic plexus of mice develops mainly after birth. This sudden increase in lymphatic vessel growth and branching is likely related to the gain of function of the diaphragm with the initiation of respiration after birth. The postnatal diaphragm provides several advantages for lymphatic development studies, as compared to other currently used model organ systems for developmental lymphangiogenesis, including the tail dermis [39] and the mesentery [27]. The diaphragm is easily accessible for dissection and for intraperitoneal manipulations, and it is well suited for whole-mount staining and imaging due to its thin and translucent structure. Moreover, as compared to the mesentery, it only needs a short handling time for whole-mount staining approaches. Based on the results of our study, combined with recent reports [14, 26], we propose five parameters that are easily quantifiable and that allow a detailed and reproducible evaluation of lymphatic vessel development in the diaphragm: the average area covered by LYVE-1+ lymphatic vessels, the number of branches, the number of vessel loops, the average branch length and the average lymphatic vessel diameter. Further advantageous features of the diaphragm model include the easy detection of lymphatic vessel sprouts, allowing detailed studies of developing lymphatic vessel tips and their interactions with other cell types such as macrophages. This model also allows the study of lymphatic vessel maturation processes, due to the presence of smooth muscle cell coverage of lymphatic vessels, the gradual downregulation of LYVE-1 expression and the formation of lymphatic valves. Importantly, lymphatic vessels in the diaphragm can also be easily studied in adult mice whereas, e.g., whole-mount stainings of mesenteric lymphatic vessels are not feasible in adult mice due to fat deposition.

Our findings that the growth of lymphatic vessels on the pleural side of the diaphragmatic muscle was almost completely inhibited in newborn K14-VEGFR-3-Fc transgenic mice, where the soluble form of VEGFR-3 acts as a decoy receptor for its ligands VEGF-C and VEGF-D, are in line with previous reports about the postnatal inhibition of lymphatic development in these mice [17, 22]. This effect seems to be transient, as lymphatic vessels were reported to be present in diaphragms of adult K14-VEGFR-3-Fc transgenic mice [22]. The important role of the VEGF-C/VEGF-D/VEGFR-3 signaling system was further supported by our finding that systemic treatment with the VEGFR-3-blocking antibody mF4 potently inhibited diaphragmatic lymphatic vessel development, as assessed at day 5 after birth.

In contrast to VEGFR-3, the role of VEGFR-2 in lymphangiogenesis has remained less well defined; however, VEGFR-2 has previously been implicated in developmental lymphangiogenesis [34]. VEGF-C can also signal, in its processed form, via VEGFR-2, which is expressed by lymphatic endothelial cells [13, 38]. Our findings that antibody-mediated VEGFR-2 blockade had no major impact on lymphatic development in the diaphragm indicate that VEGFR-2 activity is largely dispensable in this setting. This result is in line with reports that VEGF-A, the main ligand of VEGFR-2, cannot substitute for VEGF-C-induced signaling in VEGF-C-deficient embryos [16]. Since a recent study found that LYVE-1-Cre-mediated depletion of VEGFR-2 resulted in reduced lymphatic vessel density in adult ear skin [7], these data indicate that the role of VEGFR-2 in murine lymphatic vessel physiology is organ specific and/or dependent on the developmental stage. Our finding that blockade of VEGFR-1, which is not expressed by lymphatic vessels and binds VEGF-A but not VEGF-C [25], had no significant impact on lymphatic vessel development in the diaphragm is in line with the findings in VEGFR-1-deficient mice where the lymphatic vessels in the ear skin were comparable to those of WT controls [25].

Macrophages have been generally considered as important mediators of pathological lymphangiogenesis [11], but there are only a limited number of studies addressing the role of macrophages during developmental lymphangiogenesis [10, 20]. Based on our time course analysis of pre- and postnatal development of the diaphragmatic lymphatic vascular system, we found that during diaphragm development, macrophages appear earlier than lymphatic vessels, that they are closely associated with the sprouting tips of the lymphatic vessels and that they are similarly aligned as the lymphatic vessels. Thus, we hypothesized that macrophages might have an important influence on lymphatic development and patterning in the diaphragm. However, quantitative morphological and functional analyses of diaphragmatic lymphatic vessels in postnatal *Csf1r*−/− mice and in mice treated with a blocking CSF-1R antibody surprisingly did not reveal any major abnormalities of lymphatic vessel development, despite efficient macrophage depletion. Moreover, no significant changes of the lymphatic drainage were detected. Rather, macrophage depletion resulted in a subtler phenotype, which indicates that the major function of macrophages is to modulate the patterning of lymphatic vessels, and in this case to restrain lymphatic vessel branching on the pleural side of the diaphragmatic muscle by secreted factors. These effects seem to be tissue specific since no major differences in lymphatic vessel branching were detected in the mesentery and the tail dermis of *Csf1r*−/− mice, whereas altered lymphatic patterning was observed in the trachea and ear skin of postnatal mice after CSF-1R blockade [20] and in embryonic skin and yolk sacs of *Csf1r*−/− and *PU.1*−/− embryos [10]. *PU.1*−/− mice are often used as an additional model to study the role of macrophages during development. However, since these mice die soon after birth [2, 24, 33], they could not be used in the present study as the development of lymphatic vessels in the diaphragm largely occurs after birth.

Based on our findings of the close localization of macrophages at the lymphatic tips in the diaphragm and of the increased lymphatic vessel branch formation in *Csf1r*−/− mice and after CSF-1R antibody blockade, we hypothesize that macrophages might act as chaperones, regulating the branch formation of lymphatic sprouts, similar to their role during blood vessel development in the retina [8, 29]. It will be of great interest to further define the identity and functional contribution of macrophage-derived signals during developmental lymphangiogenesis in future studies.

Taken together, our results provide new information about the time points when the submesothelial, initial diaphragmatic lymphatic plexus develops and matures, and they identify major molecular and cellular drivers of this process. The developing lymphatic vessels on the pleural side of the diaphragmatic muscle can serve as a reliable and easily quantifiable in vivo model to study physiological lymphangiogenesis. This model can also help to gain more knowledge about the lymphangiogenic process and to identify and validate new targets for anti- and pro-lymphangiogenic therapies.

## Electronic supplementary material

Below is the link to the electronic supplementary material.

Supplementary material 1 (DOCX 1691 kb)

## References

[1] Abernethy NJ, Chin W, Hay JB, Rodela H, Oreopoulos D, Johnston MG (1991). Lymphatic removal of dialysate from the peritoneal cavity of anesthetized sheep. Kidney Int.

[2] Back J, Dierich A, Bronn C, Kastner P, Chan S (2004) PU.1 determines the self-renewal capacity of erythroid progenitor cells. Blood 103(10):3615–3623. doi:10.1182/blood-2003-11-4089 10.1182/blood-2003-11-4089 14739214

[3] Baluk P, Tammela T, Ator E, Lyubynska N, Achen MG, Hicklin DJ, Jeltsch M, Petrova TV, Pytowski B, Stacker SA, Yla-Herttuala S, Jackson DG, Alitalo K, McDonald DM (2005). Pathogenesis of persistent lymphatic vessel hyperplasia in chronic airway inflammation. J Clin Invest.

[4] Cecchini MG, Dominguez MG, Mocci S, Wetterwald A, Felix R, Fleisch H, Chisholm O, Hofstetter W, Pollard JW, Stanley ER (1994). Role of colony stimulating factor-1 in the establishment and regulation of tissue macrophages during postnatal development of the mouse. Development.

[5] Choi I, Chung HK, Ramu S, Lee HN, Kim KE, Lee S, Yoo J, Choi D, Lee YS, Aguilar B, Hong YK (2011). Visualization of lymphatic vessels by Prox1-promoter directed GFP reporter in a bacterial artificial chromosome-based transgenic mouse. Blood.

[6] Dai XM, Ryan GR, Hapel AJ, Dominguez MG, Russell RG, Kapp S, Sylvestre V, Stanley ER (2002). Targeted disruption of the mouse colony-stimulating factor 1 receptor gene results in osteopetrosis, mononuclear phagocyte deficiency, increased primitive progenitor cell frequencies, and reproductive defects. Blood.

[7] Dellinger MT, Meadows SM, Wynne K, Cleaver O, Brekken RA (2013). Vascular endothelial growth factor receptor-2 promotes the development of the lymphatic vasculature. PLoS ONE.

[8] Fantin A, Vieira JM, Gestri G, Denti L, Schwarz Q, Prykhozhij S, Peri F, Wilson SW, Ruhrberg C (2010). Tissue macrophages act as cellular chaperones for vascular anastomosis downstream of VEGF-mediated endothelial tip cell induction. Blood.

[9] Goldman J, Rutkowski JM, Shields JD, Pasquier MC, Cui Y, Schmokel HG, Willey S, Hicklin DJ, Pytowski B, Swartz MA (2007). Cooperative and redundant roles of VEGFR-2 and VEGFR-3 signaling in adult lymphangiogenesis. FASEB J.

[10] Gordon EJ, Rao S, Pollard JW, Nutt SL, Lang RA, Harvey NL (2010). Macrophages define dermal lymphatic vessel calibre during development by regulating lymphatic endothelial cell proliferation. Development.

[11] Harvey NL, Gordon EJ (2012). Deciphering the roles of macrophages in developmental and inflammation stimulated lymphangiogenesis. Vasc Cell.

[12] Hirakawa S, Kodama S, Kunstfeld R, Kajiya K, Brown LF, Detmar M (2005). VEGF-A induces tumor and sentinel lymph node lymphangiogenesis and promotes lymphatic metastasis. J Exp Med.

[13] Hong YK, Lange-Asschenfeldt B, Velasco P, Hirakawa S, Kunstfeld R, Brown LF, Bohlen P, Senger DR, Detmar M (2004). VEGF-A promotes tissue repair-associated lymphatic vessel formation via VEGFR-2 and the alpha1beta1 and alpha2beta1 integrins. FASEB J.

[14] Iolyeva M, Karaman S, Willrodt AH, Weingartner S, Vigl B, Halin C (2013). Novel role for ALCAM in lymphatic network formation and function. FASEB J.

[15] Jurisic G, Detmar M (2009). Lymphatic endothelium in health and disease. Cell Tissue Res.

[16] Karkkainen MJ, Haiko P, Sainio K, Partanen J, Taipale J, Petrova TV, Jeltsch M, Jackson DG, Talikka M, Rauvala H, Betsholtz C, Alitalo K (2004). Vascular endothelial growth factor C is required for sprouting of the first lymphatic vessels from embryonic veins. Nat Immunol.

[17] Karpanen T, Wirzenius M, Makinen T, Veikkola T, Haisma HJ, Achen MG, Stacker SA, Pytowski B, Yla-Herttuala S, Alitalo K (2006). Lymphangiogenic growth factor responsiveness is modulated by postnatal lymphatic vessel maturation. Am J Pathol.

[18] Kataru RP, Jung K, Jang C, Yang H, Schwendener RA, Baik JE, Han SH, Alitalo K, Koh GY (2009). Critical role of CD11b+ macrophages and VEGF in inflammatory lymphangiogenesis, antigen clearance, and inflammation resolution. Blood.

[19] Kim KE, Koh YJ, Jeon BH, Jang C, Han J, Kataru RP, Schwendener RA, Kim JM, Koh GY (2009). Role of CD11b+ macrophages in intraperitoneal lipopolysaccharide-induced aberrant lymphangiogenesis and lymphatic function in the diaphragm. Am J Pathol.

[20] Kubota Y, Takubo K, Shimizu T, Ohno H, Kishi K, Shibuya M, Saya H, Suda T (2009). M-CSF inhibition selectively targets pathological angiogenesis and lymphangiogenesis. J Exp Med.

[21] Kunstfeld R, Hirakawa S, Hong YK, Schacht V, Lange-Asschenfeldt B, Velasco P, Lin C, Fiebiger E, Wei X, Wu Y, Hicklin D, Bohlen P, Detmar M (2004). Induction of cutaneous delayed-type hypersensitivity reactions in VEGF-A transgenic mice results in chronic skin inflammation associated with persistent lymphatic hyperplasia. Blood.

[22] Makinen T, Jussila L, Veikkola T, Karpanen T, Kettunen MI, Pulkkanen KJ, Kauppinen R, Jackson DG, Kubo H, Nishikawa S, Yla-Herttuala S, Alitalo K (2001). Inhibition of lymphangiogenesis with resulting lymphedema in transgenic mice expressing soluble VEGF receptor-3. Nat Med.

[23] Martinez-Corral I, Makinen T (2013). Regulation of lymphatic vascular morphogenesis: implications for pathological (tumor) lymphangiogenesis. Exp Cell Res.

[24] McKercher SR, Torbett BE, Anderson KL, Henkel GW, Vestal DJ, Baribault H, Klemsz M, Feeney AJ, Wu GE, Paige CJ, Maki RA (1996). Targeted disruption of the PU.1 gene results in multiple hematopoietic abnormalities. EMBO J.

[25] Murakami M, Zheng Y, Hirashima M, Suda T, Morita Y, Ooehara J, Ema H, Fong GH, Shibuya M (2008). VEGFR1 tyrosine kinase signaling promotes lymphangiogenesis as well as angiogenesis indirectly via macrophage recruitment. Arterioscler Thromb Vasc Biol.

[26] Negrini D, Moriondo A (2013). Pleural function and lymphatics. Acta Physiol.

[27] Norrmen C, Ivanov KI, Cheng J, Zangger N, Delorenzi M, Jaquet M, Miura N, Puolakkainen P, Horsley V, Hu J, Augustin HG, Yla-Herttuala S, Alitalo K, Petrova TV (2009). FOXC2 controls formation and maturation of lymphatic collecting vessels through cooperation with NFATc1. J Cell Biol.

[28] Oliver G, Srinivasan RS (2010). Endothelial cell plasticity: how to become and remain a lymphatic endothelial cell. Development.

[29] Rymo SF, Gerhardt H, Wolfhagen Sand F, Lang R, Uv A, Betsholtz C (2011). A two-way communication between microglial cells and angiogenic sprouts regulates angiogenesis in aortic ring cultures. PLoS ONE.

[30] Sabine A, Petrova TV (2014). Interplay of mechanotransduction, FOXC2, connexins, and calcineurin signaling in lymphatic valve formation. Adv Anat Embryol Cell Biol.

[31] Schulte-Merker S, Sabine A, Petrova TV (2011). Lymphatic vascular morphogenesis in development, physiology, and disease. J Cell Biol.

[32] Schulz MM, Reisen F, Zgraggen S, Fischer S, Yuen D, Kang GJ, Chen L, Schneider G, Detmar M (2012). Phenotype-based high-content chemical library screening identifies statins as inhibitors of in vivo lymphangiogenesis. Proc Natl Acad Sci USA.

[33] Scott EW, Simon MC, Anastasi J, Singh H (1994). Requirement of transcription factor PU.1 in the development of multiple hematopoietic lineages. Science.

[34] Secker GA, Harvey NL (2015). VEGFR signaling during lymphatic vascular development: from progenitor cells to functional vessels. Dev Dyn.

[35] Shibata SJ, Hiramatsu Y, Kaseda M, Chosa M, Ichihara N, Amasaki H, Hayakawa T, Asari M (2006). The time course of lymph drainage from the peritoneal cavity in beagle dogs. J Vet Med Sci.

[36] Sudo T, Nishikawa S, Ogawa M, Kataoka H, Ohno N, Izawa A, Hayashi S (1995). Functional hierarchy of c-kit and c-fms in intramarrow production of CFU-M. Oncogene.

[37] Tammela T, Alitalo K (2010). Lymphangiogenesis: molecular mechanisms and future promise. Cell.

[38] Wirzenius M, Tammela T, Uutela M, He Y, Odorisio T, Zambruno G, Nagy JA, Dvorak HF, Yla-Herttuala S, Shibuya M, Alitalo K (2007). Distinct vascular endothelial growth factor signals for lymphatic vessel enlargement and sprouting. J Exp Med.

[39] Xu Y, Yuan L, Mak J, Pardanaud L, Caunt M, Kasman I, Larrivee B, Del Toro R, Suchting S, Medvinsky A, Silva J, Yang J, Thomas JL, Koch AW, Alitalo K, Eichmann A, Bagri A (2010). Neuropilin-2 mediates VEGF-C-induced lymphatic sprouting together with VEGFR3. J Cell Biol.

[40] Yang Y, Oliver G (2014). Development of the mammalian lymphatic vasculature. J Clin Invest.

[41] Yuen D, Pytowski B, Chen L (2011). Combined blockade of VEGFR-2 and VEGFR-3 inhibits inflammatory lymphangiogenesis in early and middle stages. Invest Ophthalmol Vis Sci.

[42] Zumsteg A, Baeriswyl V, Imaizumi N, Schwendener R, Rüegg C, Christofori G (2009). Myeloid cells contribute to tumor lymphangiogenesis. PLoS ONE.

