# Supplementary material for: Regulation of lymphangiogenesis in the diaphragm by macrophages and VEGFR-3 signaling
Source: Angiogenesis. 2016 Jul 27;19(4):513–24. doi: 10.1007/s10456-016-9523-8 (PMC5026726; doi:10.1007/s10456-016-9523-8)

**Supplementary Figure Legend**

**Fig. S1. CSF-1R-driven macrophage depletion does not influence mesenteric and tail dermis lymphatic vessel development.**

Macrophage depletion was confirmed by CD206 (cyan) and CD68 (red) stained whole mounts of tail skins of P7 WT **(A)** and *Csf1r*-/- littermates **(B).** CD11b stainings of frozen sections of P7 WT **(C)** and *Csf1r*-/- **(D)** littermates showed macrophage depletion in back skin. LYVE-1 whole mounts of tail skin of WT **(E)** and *Csf1r*-/- **(F)** littermates showed no obvious difference. Quantifications of tail dermis lymphatic vessel development of *Csf1r*-/- pups showed no significant changes in LYVE-1 area **(G)** and branches per ring structure **(H)** compared to the WT control. CD31 (red) and PROX-1 (green) whole mounts of mesenteries of WT **(I)** and *Csf1r*-/- littermates **(J)**. Quantification of mesenteric lymphatic vessel development of *Csf1r*-/- pups showed no significant changes in branch and valve counts compared to the WT control **(K, L)**. Macrophage depletion was demonstrated by F4/80 stained diaphragm whole mounts of P7 IgG **(M)** and AFS98 **(N)** treated pups. F4/80 stainings of frozen sections of P7 IgG **(O)** and AFS98 **(P)** treated pups show macrophage depletion in back skin. Scale bars: (A)-(D): 50 µm, (E)-(J): 100 µm, and (M)-(P): 50 µm.

**Supplementary Fig. 1**


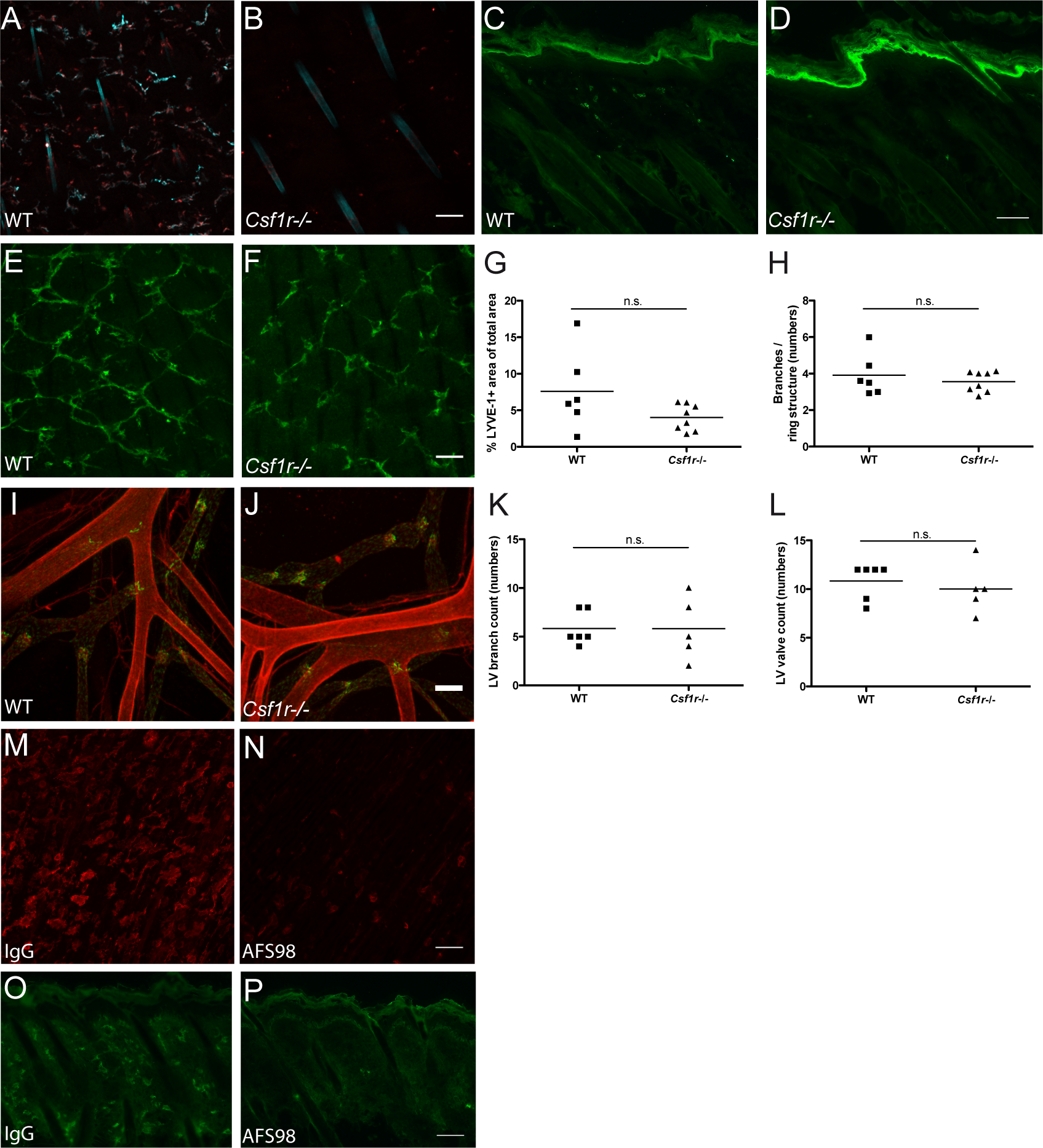

Supplement: Supplementary file 1 — Supplementary material 1 (DOCX 1691 kb) [file 10456_2016_9523_MOESM1_ESM.docx]
